# Knockdown of ILK Alleviates High Glucose-Induced Damage of H9C2 Cells through TLR4/MyD88/NF-*κ*B Pathway

**DOI:** 10.1155/2022/6205190

**Published:** 2022-05-05

**Authors:** Qiang Yin, Zhendong Li, Shaoying Lu

**Affiliations:** ^1^Department of Vascular Surgery, The First Affiliated Hospital of Xi'an Jiaotong University, Xi'an, China; ^2^Department of Cardiovascular Surgery, Second Affiliated Hospital, Xi'an Medical University, Xi'an, China; ^3^Department of Vascular Surgery, Wuxi Mingci Cardiovascular Hospital, Wuxi, China

## Abstract

The aim of this study was to explore the role of ILK in an in vitro model of diabetic cardiomyopathy. We used 30 mmol/L high glucose to treat H9C2 cells to construct an in vitro model, knocked down the ILK expression level of H9C2 cells by small interference technology, and detected the activity of antioxidant enzymes and inflammatory factors in the supernatant. The expression levels of SOD1 and IL-1*β* were detected by immunofluorescence staining. The expression levels of the TLR4/MyD88/NF-*κ*B signaling pathway and its downstream factors were detected by quantitative reverse-transcription polymerase chain reaction (qRT-PCR). Compared with the control group, after high-glucose culture of H9C2 cells, the cell activity decreased, while the apoptosis rate increased, with the TLR4/MyD88/NF-*κ*B signaling pathway activated, thereby inducing oxidative stress and inflammation. Compared with the high-glucose group, the HG+si-ILK group increased cell activity, decreased the apoptosis rate, and inhibited the excessive activation of the TLR4/MyD88/NF-*κ*B signaling pathway, thereby improving oxidative stress and inflammation. Knockdown of ILK expression can protect H9C2 cells from reducing high glucose-induced inflammation, oxidative stress, and apoptosis by inhibiting the TLR4/MyD88/NF-*κ*B signaling pathway.

## 1. Introduction

With the changes in lifestyle and diet structure, the incidence of metabolic diseases, especially obesity and type 2 diabetes, has increased year by year globally. Diabetic cardiomyopathy (DCM) has become one of the most important diseases that threaten human health [1]. DCM was first proposed by Rubler in 1972 [2]. It refers to myocardial diseases that occur in diabetic patients and cannot be explained by hypertension, coronary heart disease, heart valve disease, and other heart diseases. The structural change is the main performance, which can eventually progress to heart failure (HF) [3]. Since then, research on DCM has been carried out one after another and has achieved certain results, but its pathogenesis has not yet been fully elucidated. Previous evidence showed that metabolic disorders, oxidative stress (OS), inflammatory response, and apoptosis all played crucial roles in the pathogenesis of DCM [4–6]. Diabetes is an independent risk factor for cardiovascular disease (CVD). Patients with CVD and diabetes have worse short-term or long-term prognosis than those without diabetes. Therefore, exploring the pathogenesis of DCM is particularly important.

Integrin-linked kinase (ILK) is a serine-threonine protein kinase in the lysates of animal epithelial cells and tumor cells. As a crucial linker protein and signaling protein, integrin and growth factor receptors play a key role in mediating the interaction between cells and extracellular matrix and signal transmission inside and outside cells [7]. ILK is most abundantly distributed in the heart. Studies have found that ILK may play a role as a regulator in the cardiac hypertrophy signal transduction pathway [8]. ILK has a nonnegligible effect on the occurrence and development of cardiomyopathy. Therefore, we speculate that ILK might play a crucial role in regulating high glucose-induced cardiomyocyte apoptosis, OS, and inflammatory response.

Toll-like receptors (TLRs) are a family of receptors that mediate innate immunity. As a key transmembrane protein that triggers inflammation, it is involved in the regulation of immune inflammation and is considered to be the main body that mediates immune and inflammation [9]. Studies have shown that TLRs not only recognize the exogenous ligand lipopolysaccharide but also the endogenous ligand expressed during diabetes injury and play a major role in the occurrence and development of diabetes and its complications [10]. TLR4 is the first TLRs found in mammals. After TLR4 on the surface of myocardial cell membrane is stimulated by the corresponding ligand, it can activate the immune inflammation of myocardial tissue [11]. TLR4 can activate MAPK and NF-*κ*B signaling pathways through myeloid differentiation factor 88- (MyD88-) dependent pathway cascade reactions, thus promoting the expression of related genes and initiating natural immunity [12]. NF-*κ*B is a crucial nuclear transcription factor, located in the pivotal position of the signaling pathway downstream of TLRs. It can complete the transmission of inflammation signals by regulating the cascade of immune and inflammation-related factors and inflammatory transmitters [13]. Therefore, the TLR4-MyD88-NF-*κ*B signaling pathway plays a crucial pivotal role in the regulation of immune and inflammatory responses. At present, the mechanism of TLR4-MyD88-NF-*κ*B signaling pathway mediating inflammatory response in diabetic nephropathy and vascular disease is relatively clear, and the specific mechanism of its regulation and control of DCM remains to be further studied.

## 2. Materials and Method

### 2.1. Cell Culture

H9C2 cells were purchased from Shanghai Institute of Life Sciences, Chinese Academy of Sciences (Cell Culture Center, Shanghai, China), and 3 to 8 passage cells were selected for experiment. H9C2 cells were cultured in Dulbecco's Modified Eagle's Medium (DMEM; Life Technology, Wuhan, China) containing 10% fetal bovine serum (FBS; Life Technology, Wuhan, China) in a 37°C normoxic cell incubator, and the fluid was changed every other day.

### 2.2. Cell Transfection and Treatment

H9C2 cells at log phase were planted in 6-well plates. Transfection should be performed after the cells adhere to the wall, and the confluence of the cells should be 30%-50% during transfection. Then, we transfected H9C2 cells with si-ILK and si-NC according to the instructions of Lipofectamine^2000^ Transfection Reagent (Thermo Fisher Scientific, Waltham, MA, USA) and observed the cell status under an optical microscope (Becton Dickinson, Heidelberg, Germany). And 24 hours after transfection, the cell culture medium was changed.

### 2.3. Western Blot (WB)

H9C2 cells were cultured in 6-well plates and were divided into different groups as mentioned above. When the cell fusion degree is about 80%, 50 *μ*L of lysate (Beyotime, Nanjing, China) was used to lyse the cultured H9C2 cells. Protein concentration detection kit (Beyotime, Nanjing, China) was used to detect the concentration of the extracted protein. After boiling the protein sample, 40 *μ*g protein sample was added into each well for the electrophoresis at a voltage of 90 V. Then, the protein was transferred to the membrane at a constant current of 250 mA for 2 hours. Then, the polyvinylidene difluoride (PVDF, Thermo Fisher Scientific, Waltham, MA, USA) membranes were blocked with 5% skimmed milk powder, followed by being incubated with the primary antibodies (IL-1*β*, Abcam, 1 : 1000, Cambridge, MA, USA) overnight at 4°C. After washing with phosphate-buffered saline (PBS) buffer, the secondary antibody labeled with horseradish peroxidase was added to the membranes for incubation at room temperature. After incubation for 1 hour, the membranes reacted with electrochemiluminescence (ECL, Yifei Xue Biotechnology, Nanjing, China) and were exposed in the dark room.

### 2.4. RNA Isolation and Quantitative Reverse-Transcription Polymerase Chain Reaction (qRT-PCR)

Prechilled PBS was used to wash H9C2 cells, and then, TRIzol reagent (Thermo Fisher Scientific, Waltham, MA, USA) was used to extract total cellular RNA. The reverse transcription kit (Thermo Fisher Scientific, Waltham, MA, USA) was used to transcribe RNA into cDNA. RT-PCR was performed according to a previous report [12]. Primers used are shown in Table 1.

### 2.5. MTT Assay

Trypsin (Kaiji, Nanjing, China) was used to digest H9C2 cells, and then, cells were formulated into a cell suspension with a concentration of 1 × 10^5^/mL. Then, the cells were inoculated into 96-well plates at 1 × 10^4^ cells/well with the total amount of medium 100 *μ*L per well. Then, the cells were incubated in a 37°C, 5% CO_2_ incubator. Next, different stimulations were given to each experimental group. After incubating for 0, 4, 8, 12, 16, 20, and 24 hours, 20 *μ*L of MTT solution (Construction, Nanjing, China) was added to each well. After 4 hours, the culture was terminated and 150 *μ*L of dimethyl sulfoxide (Construction, Nanjing, China) was added to each well after discarding the supernatant. Finally, a microplate reader was used to measure OV570 value, and the cell survival rate was calculated according to the formula.

### 2.6. Tunel Staining

When the cells were nearly 80% confluent, 4% paraformaldehyde (Construction, Nanjing, China) was used for the fixation. Then, PBS was used to wash the cells to remove excess paraformaldehyde. Each group of cells was stained according to the instructions of the Tunel kit (Kaiji, Nanjing, China). 4′,6-Diamidino-2-phenylindole (DAPI, Construction, Nanjing, China) was used to stain the nucleus; the positive cells were observed under a fluorescent microscope (Becton Dickinson, Heidelberg, Germany).

### 2.7. Enzyme-Linked Immunosorbent Assay (ELISA)

After collecting the cell supernatant of each group, ELISA was performed according to the instructions of the kit (Elabscience, Wuhan, China). 100 *μ*L of biotinylated antibody was added to each well for incubation at 37°C. After 1 hour, 100 *μ*L of enzyme-binding working solution was added to each well. After the incubation was completed, the color developing agent and the terminating agent were added, and then, the OD450 value was measured with a microplate reader (Becton Dickinson, Heidelberg, Germany).

### 2.8. Determination of Malondialdehyde (MDA) Levels, Superoxide Dismutase (SOD), Catalase (CAT), and Glutathione Peroxidase (GSH-Px) Activity in Cell Supernatants

According to the instructions of the above kits (Construction, Nanjing, China), the cell culture supernatant or cell lysate of each group was collected after 24 hours of culture. Then, we used a microplate reader to detect the absorbance of each sample at different wavelengths and calculated according to the corresponding formula.

### 2.9. Immunofluorescence Staining

When the cells were nearly 80% confluent, 4% paraformaldehyde was used to fix the cells, then PBS was used to wash the cells to remove excess paraformaldehyde, and then, 50 ml/L goat serum was used to block for 30 minutes. After decanting excess serum, the primary antibodies SOD1 (Abcam, Cambridge, MA, USA, Rabbit, 1 : 1000) and IL-1*β* (Abcam, Cambridge, MA, USA, Rabbit, 1 : 1000) were used for incubation at 4°C overnight. After washing with PBS, the secondary antibody was added dropwise and incubated at room temperature in the dark for 60 min. DAPI was used to stain the nuclei for 10 minutes. Finally, the results were observed and recorded under a fluorescent microscope.

### 2.10. Statistical Analysis

Statistical Product and Service Solutions (SPSS) 21.0 (SPSS IBM, Armonk, NY USA) statistical software was used to analyze the experimental data. Measurement data were expressed as *χ* ± *s*. The *t*-test was used for comparisons between the two groups. Comparison between multiple groups was done using one-way analysis of variance (ANOVA) test followed by a post hoc test (Least Significant Difference). The Least Significant Difference (LSD) test or Student-Newman-Keuls (SNK) test was used for pairwise comparison under the condition of homogeneity of variance. Test level *α* = 0.05. All experiments were repeated 3 times.

## 3. Results

### 3.1. High Glucose Damages H9C2 Cells

**Figure 1:**
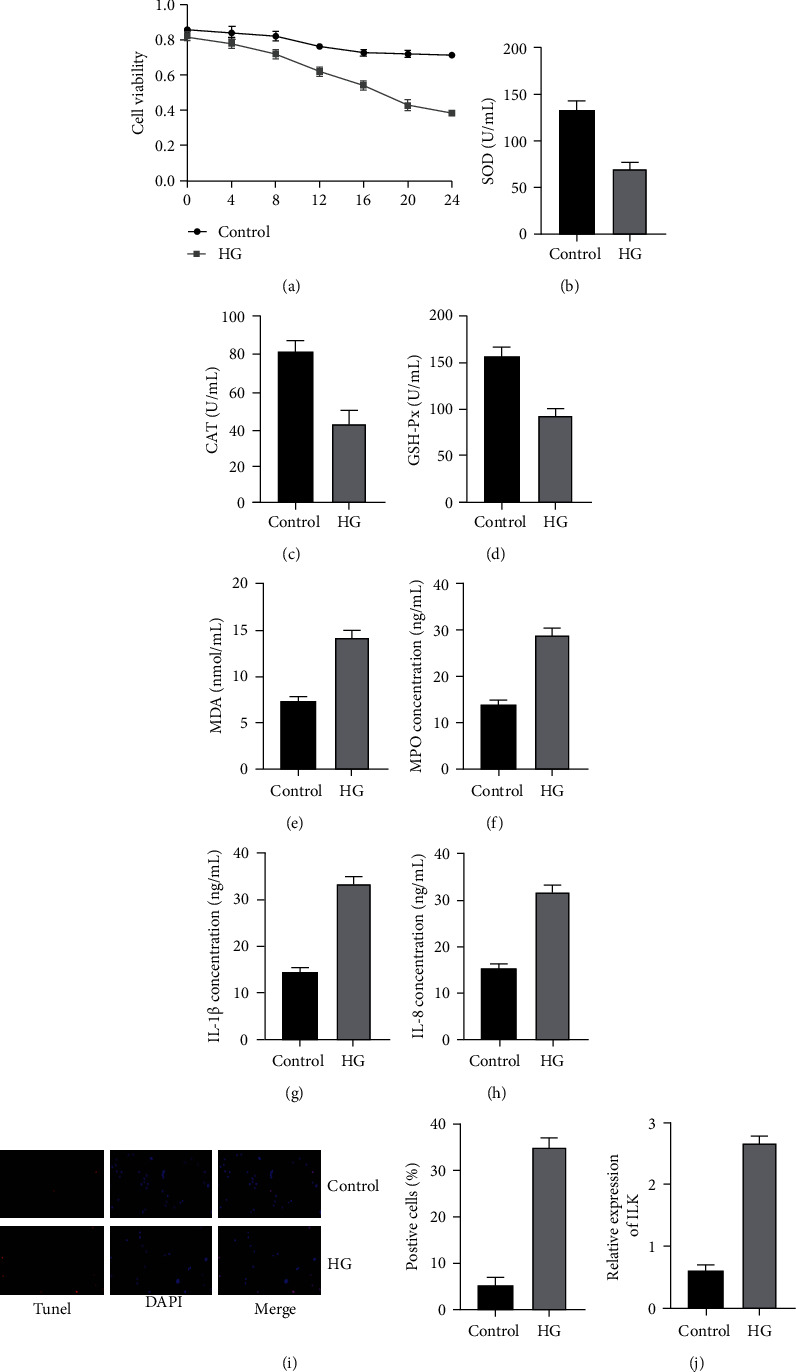
High glucose damages H9C2 cells. (a) The cell activity of H9C2 was determined by MTT assay. (b–e) The levels of SOD, CAT, GSH-Px, and MDA in the supernatant were determined by the kits. (f–h) The levels of MPO, IL-1*β*, and IL-8 in the supernatant were determined by the ELISA. (i) Tunel staining was performed to examine the apoptosis level of H9C2. (j) mRNA expression results of ILK were determined by real-time PCR. (“_∗_” indicates statistical difference from the control group *P* < 0.05.).

According to reports in the literature, we selected 30mmol/L high glucose to treat H9C2 cells and tested the cell activity of each group by MTT test (Figure 1(a)). The results confirmed that high glucose can reduce the activity of H9C2 cells, and as the culture time was delayed, the lower the activity. Subsequently, we used the kit to detect the antioxidant enzyme activity in the cell supernatant of each group (Figures 1(b)–1(d)). It was found that the activities of SOD, CAT, and GSH-Px in the high-glucose group were dramatically reduced. At the same time, the MDA content also increased dramatically (Figure 1(e)). In addition, we detected the content of inflammatory factors in the supernatant of each group by ELISA method (Figures 1(f)–1(h)). The results showed that the levels of MPO, IL-1*β*, and IL-8 in the high-glucose group increased dramatically. In addition, Tunel staining also found that the apoptosis rate in the high-glucose group was dramatically increased (Figure 1(i)). In summary, we verified that high glucose can induce H9C2 cells to produce OS and inflammatory responses, thereby promoting apoptosis. And RT-PCR results showed that ILK mRNA in the high-glucose group was dramatically higher than that in the control group (Figure 1(j)). Given this, we speculated that ILK was involved in the process of high-glucose damage to H9C2 cells.

### 3.2. Knockdown of ILK Improves H9C2 Oxidative Stress Induced by High Glucose

**Figure 2:**
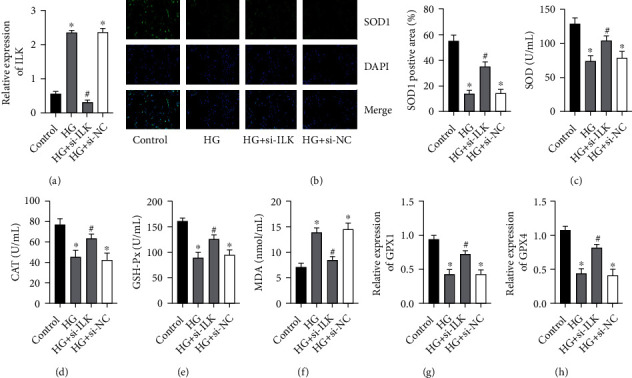
Knockdown of ILK improves H9C2 oxidative stress induced by high glucose. (a) mRNA expression results of ILK were determined by real-time PCR. (b) Immunofluorescence was performed to examine the expression of SOD1. (c–f) The levels of SOD, CAT, GSH-Px, and MDA in the supernatant were determined by the kits. (g, h) mRNA expression results of GPX1 and GPX4 were determined by real-time PCR. (“_∗_” indicates statistical difference from the control group *P* < 0.05, and “#” indicates statistical difference from the HG group *P* < 0.05.).

Next, we used small interference technology to knock down the ILK expression level of H9C2 cells and verified our transfection efficiency by RT-PCR (Figure 2(a)). First, we detected the expression level of SOD1 in 4 groups by immunofluorescence staining and found that the expression of SOD1 in the high-glucose group was lower than that in the control group, and after knocking down the expression of ILK, the expression level of SOD1 increased dramatically (Figure 2(b)). There was no significant difference in the HG+si-NC group. Next, we detected SOD, CAT, and GSH-Px activity and MDA content in the supernatant (Figures 2(c)–2(f)). The results showed that the activities of SOD, CAT, and GSH-Px in the HG+si-ILK group were dramatically increased. At the same time, the MDA content was dramatically reduced. In addition, RT-PCR detection of GPX1 and GPX4 mRNA expression also found that the high-glucose group GPX1 and GPX4 mRNA expression levels were dramatically reduced, while in the HG+si-ILK group, GPX1 and GPX4 mRNA expression levels were increased (Figures 2(g) and 2(h)). There was no significant difference in the HG+si-NC group.

### 3.3. Knocking Down ILK Can Improve H9C2 Inflammatory Response Induced by High Glucose

**Figure 3:**
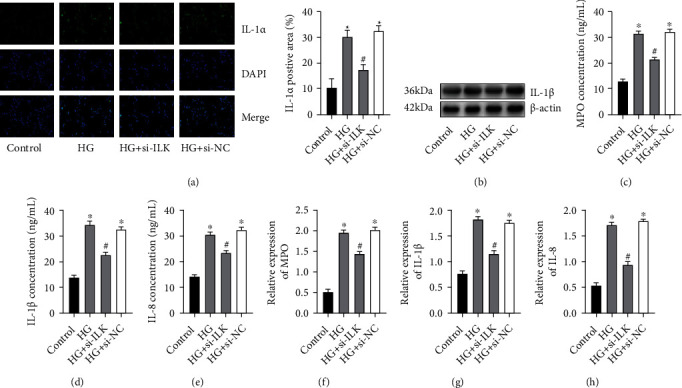
Knocking down ILK can improve H9C2 inflammatory response induced by high glucose. (a) Immunofluorescence was performed to examine the expression of IL-*α*. (b) Protein expression results of IL-1*β* were determined by Western blotting. *β*-Actin was used as an internal control. (c–e) The levels of MPO, IL-1*β*, and IL-8 in the supernatant were determined by the ELISA. (f–h) mRNA expression results of MPO, IL-1*β*, and IL-8 were determined by real-time PCR. (“_∗_” indicates statistical difference from the control group *P* < 0.05, and “#” indicates statistical difference from the HG group *P* < 0.05.).

The expression level of IL-*α* in the 4 groups was detected by immunofluorescence staining (Figure 3(a)). It was found that the expression of IL-*α* in the high-glucose group was higher than that in the control group, but after knocking down the expression of ILK, the expression level of IL-*α* was dramatically reduced. There was no significant difference in the HG+si-NC group. Next, WB results also found that the expression of IL-1*β* protein in the high-glucose group was dramatically higher than that in the control group, while the expression of IL-1*β* protein in the HG+si-ILK group was dramatically reduced, and there was no significant difference in the results of the HG+si-NC group (Figure 3(b)). Subsequently, the content of MPO, IL-1*β*, and IL-8 in the supernatant was detected by the ELISA method (Figures 3(c)–3(e)). The content of the above inflammatory factors in the HG+si-ILK group was dramatically reduced, and there was no significant difference in the results of the HG+si-NC group. At the same time, RT-PCR also obtained similar results to the former (Figures 3(f)–3(h)).

### 3.4. Knockdown of ILK Can Inhibit High Glucose-Induced H9C2 Apoptosis and Inhibit TLR4/MyD88/NF-*κ*B Pathway Activation

**Figure 4:**
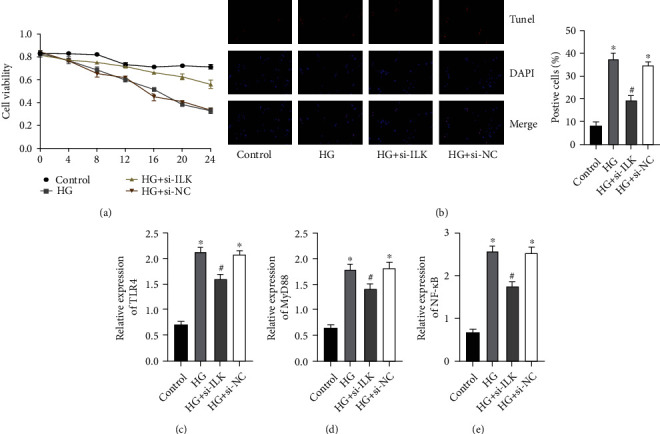
Knockdown of ILK can inhibit high glucose-induced H9C2 apoptosis and inhibit TLR4/MyD88/NF-*κ*B pathway activation. (a) The cell activity of H9C2 was determined by MTT assay. (b) Tunel staining was performed to examine the apoptosis level of H9C2. (c–e) mRNA expression results of TLR4, MyD88, and NF-*κ*B were determined by real-time PCR. (“_∗_” indicates statistical difference from the control group *P* < 0.05, and “#” indicates statistical difference from the HG group *P* < 0.05.).

First, we tested the cell viability of the 4 groups by MTT method (Figure 4(a)). The results found that when we knocked down ILK expression, the activity of H9C2 cells was higher than that of the HG group. Compared with the HG group, there was no significant difference in the results of the HG+si-NC group. And Tunel staining also found that in the HG+si-ILK group, the apoptosis rate induced by high glucose was also dramatically reduced, and there was no significant difference in the results of the HG+si-NC group (Figure 4(b)). Next, we detected TLR4, MyD88, and NF-*κ*B mRNA levels by RT-PCR (Figures 4(c)–4(e)). The results showed that after H9C2 cells were cultured in high glucose, TLR4, MyD88, and NF-*κ*B mRNA were dramatically increased, while in the HG+si-ILK group, TLR4, MyD88, and NF-*κ*B mRNA were dramatically inhibited. Compared with the HG group, there was no significant difference in the results of the HG+si-NC group. The above results indicated that knocking down ILK expression can dramatically inhibit high glucose-induced overactivation of the TLR4-MyD88-NF-*κ*B signaling pathway in H9C2 cells and inhibit H9C2 cell apoptosis.

## 4. Discussion

Diabetes is one of the important chronic noninfectious diseases that seriously threaten human health in today's society, and it shows a significant growth trend worldwide. DCM is an independent diabetic cardiovascular complication. It is a special form of heart disease. Its early stage is mainly diastolic dysfunction. With the extension of time, the contractile function is damaged, and the myocardium is extensively necrotic. Decreased blood fraction can eventually lead to cardiogenic shock, HF, and even death [14]. DCM is mainly due to a series of pathological changes such as inflammatory response of myocardial cells caused by diabetic metabolic dysfunction, redox imbalance, myocardial cell necrosis, interstitial fibrosis, and myocardial cell hypertrophy [15]. Therefore, we constructed a DCM model in vitro to explore its pathogenesis.

First, we verified the success of the construction of an in vitro model of DCM by measuring cell activity, antioxidant enzyme activity, inflammatory factor expression levels, and apoptosis rates, and our results are consistent with previous studies [16]. Then, we found that in the high-glucose group, ILK expression was dramatically increased. Therefore, we speculate that ILK is involved in regulating the occurrence and development of DCM. ILK, as a key regulator of cellular response integrins and growth factor-mediated signals, is involved in the formation of cytoskeleton, cell proliferation, apoptosis, and maintenance of oxygen balance, as well as the regulation of inflammatory factors [17–19]. From this, we speculate that ILK plays a crucial role in regulating high glucose-induced H9C2 cell OS, inflammation, and apoptosis. Immediately afterwards, we first knocked down ILK expression levels through small interference techniques. By detecting the activity of antioxidant enzymes in the cell supernatant, we found that when we knocked down the expression of ILK, the activities of SOD, CAT, and GSH-Px increased dramatically, and the MDA content also decreased. MDA is one of the most important products of membrane lipid peroxidation, and its content can affect the mitochondrial respiratory chain complex and key enzyme activity in the mitochondria [20]. Therefore, we can understand the degree of OS damage in cells by detecting MDA content. In addition, high glucose reduced the expression levels of SOD1, GPX1, and GPX4. After ILK knockdown, the expression levels of SOD1, GPX1, and GPX4 increased dramatically. Based on the above results, we confirmed that knocking down ILK expression can improve high glucose-induced redox imbalance.

Inflammatory response plays a crucial regulatory role in the pathogenesis of DCM. At the same time, inflammation is a crucial part of the immune system, triggered by any stimulus that threatens tissue homeostasis. TLRs, as key transmembrane proteins that trigger inflammatory responses, participate in the regulation of immune inflammatory responses and are considered to be the main body mediating immune and inflammatory responses. After binding to specific ligands, TLRs activate the NF-kB signaling pathway through the MyD88-dependent pathway, promote the expression of related genes, and induce inflammatory response. Therefore, the effective intervention of inflammation in the early stage of diabetes can inhibit the expression of inflammatory factors in the blood circulation and local heart and reduce the infiltration of immune active cells into the heart muscle, thereby reducing the heart inflammation and improving cardiac dysfunction [21, 22]. Our results found that high glucose can activate the TLR4/MyD88/NF-*κ*B signaling pathway, thereby upregulating the expression of downstream inflammatory factors. When we knocked down ILK expression, the TLR4/MyD88/NF-KB signaling pathway was suppressed, the downstream inflammatory factor expression level was also suppressed, and the apoptosis of H9C2 cells was also decreased. Thus, we confirmed that knocking down ILK expression can inhibit high glucose-induced TLR4/MyD88/NF-KB signaling pathway overactivation, thereby inhibiting downstream inflammatory factor expression, and increase the H9C2 activity.

## 5. Conclusion

Taken above, knocking down ILK expression can improve OS and inflammation through the TLR4/MyD88/NF-*κ*B signaling pathway, thereby reducing the apoptosis rate of H9C2 cells. These findings may provide a new target for the treatment of DCM.

## Figures and Tables

**Table 1:** Primer sequences of quantitative reverse transcription-polymerase chain reaction.

Oligo name	Sequence (5′ → 3′)	
GPX1 (rat)	Forward	ATCATATGTGTGCTGCTCGGCTAGC
	Reverse	TACTCGAGGGCACAGCTGGGCCCTTGAG
GPX4 (rat)	Forward	GGACCTGCCGTGCTATCT
	Reverse	GGCCTCTGGACCTTCCTC
ILK (rat)	Forward	ATGGCTTCTCCCCTTTG
	Reverse	GTATCATCCCCACGATTCA
IL-1*β* (rat)	Forward	GCAACTGTTCCTGAACTCAACT
	Reverse	ATCTTTTGGGGTCCGTCAACT
IL-8 (rat)	Forward	CAAGGCTGGTCCATGCTCC
	Reverse	TGCTATCACTTCCTTTCTGTTGC
MPO (rat)	Forward	CTGGCACGGAAGCTGAT
	Reverse	AATGAGGCAGGCAAGGAG
TLR4 (rat)	Forward	GACTCCATTCAAGCCCAA
	Reverse	TCTCCCAAGATCAACCGA
MyD88 (rat)	Forward	TGAGCAACCAGGACAGC
	Reverse	TAGGCATGTCAGGGGAGA
NF-kB (rat)	Forward	ACTGCCGGGATGGCTACTAT
	Reverse	TCTGGATTCGCTGGCTAATGG
GAPDH (rat)	Forward	CAACTCCCTCAAGATTGTCAGCAA
	Reverse	GGCATGGACTGTGGTCATGA

## Data Availability

The datasets used and analyzed during the current study are available from the corresponding author on reasonable request.
